# Supplementary material for: MALDI-TOF MS Limits for the Identification of Mediterranean Sandflies of the Subgenus Larroussius, with a Special Focus on the Phlebotomus perniciosus Complex
Source: Microorganisms. 2022 Oct 28;10(11):2135. doi: 10.3390/microorganisms10112135 (PMC9698236; doi:10.3390/microorganisms10112135)
Supplement: Supplementary file 1 [file microorganisms-10-02135-s001.zip › microorganisms-1879145-supplementary.pdf]

Supplementary Table S1: Results of Reims and Marseille MSP database for identification

		Reims MSP database					Marseille MSP database				
**Analyte**	**Species**	**First match**	**LSV1**	**Second match**	**LSV2**	**Species present in database**	**First match**	**LSV1**	**Second match**	**LSV2**	**Species present in database**
AM_115	*Sergentomyia minuta*	**unreliable identification**	0.52	**unreliable identification**	0.28	No	*Sergentomyia minuta*	1.22	*Sergentomyia minuta*	1.1	Yes
AM_119	*Ph. perniciosus (PNA)*	PT19 *Ph perniciosus* female (PN)	1.8	MOF10 *Ph perniciosus* male (PNA)	1.47	Yes	*Ph. perniciosus* male	1.26	*Ph. perniciosus* male	1.23	Yes
AM_131	*Sergentomyia fallax*	**unreliable identification**	0.74	**unreliable identification**	0.51	No	*Sergentomyia minuta*	1.13	*Phlebotomus sergenti*	1.02	Yes
AM_228	*Ph. longicuspis*	PT19 *Ph perniciosus* female (PN)	1.71	ES08 *Ph perniciosus* female (PN)	1.21	No	*Ph. perfiliewi*	1.09	*Ph. perniciosus female*	1.06	Yes
AM_229	*Sergentomyia fallax*	**unreliable identification**	0.41	**unreliable identification**	0.31	No	*Sergentomyia minuta*	1.01	**unreliable identification**	0.84	No
AM_239	*Ph. papatasi*	**unreliable identification**	0.75	**unreliable identification**	0.72	Yes	*Ph. perniciosus* female	1.05	**unreliable identification**	0.99	Yes
AM_243	*Ph. longicuspis*	PT19 *Ph perniciosus* female (PN)	1.72	MOZ14 *Ph longicuspis* female	1.61	Yes	*Ph. longicuspis* female	1.5	*Ph. longicuspis* male	1.48	Yes
AM_245	*Ph. longicuspis*	PT19 *Ph perniciosus* female (PN)	1.63	MOZ3 *longicuspis* female	1.58	Yes	*Ph. longicuspis* female	1.49	*Ph. longicuspis* male	1.38	Yes
AM_332	*Sergentomyia fallax*	**unreliable identification**	0.82	**unreliable identification**	0.25	No	*Sergentomyia minuta*	1.07	**unreliable identification**	0.91	No
AM_342	*Ph. papatasi*	ES41 Ph. papatasi (male)	1.36	ES08 *Ph perniciosus* female (PN)	0.76	Yes	**unreliable identification**	0.89	**unreliable identification**	0.84	Yes
AM_352	*Ph. papatasi*	ES41 Ph. papatasi (male)	1.72	PT31 *Ph perniciosus* male (PN)	1.14	Yes	*Ph. perfiliewi*	1.05	*Ph. perniciosus* male	1.03	Yes
AM_380	*Ph. perniciosus (PNA)*	PT19 *Ph perniciosus* female (PN)	1.62	MOF10 *Ph perniciosus* male (PNA)	1.28	Yes	*Ph. perniciosus* male	1.35	*Ph. perniciosus* male	1.29	Yes

AM_390	*Sergentomyia minuta*	**unreliable identification**	0.79	**unreliable identification**	0.78	No	*Sergentomyia minuta*	1.46	*Sergentomyia minuta*	1.02	Yes
AM_433	*Ph. sergenti*	**unreliable identification**	0.75	**unreliable identification**	0.74	No	*Ph. perniciosus female*	1.01	**unreliable identification**	0.94	Yes
AM_434	*Ph. sergenti*	**unreliable identification**	0.88	**unreliable identification**	0.8	No	**unreliable identification**	0.9	**unreliable identification**	0.85	Yes
AM_536	*Ph. sergenti*	**unreliable identification**	0.93	**unreliable identification**	0.71	No	**unreliable identification**	0.91	**unreliable identification**	0.84	Yes
AM_586	*Ph. perniciosus (PNA)*	PT19 *Ph perniciosus* female (PN)	1.75	MT06 *Ph perniciosus* female (PN)	1.38	Yes	*Ph. perniciosus* male	1.32	*Ph. perniciosus* male	1.18	Yes
AM_622	*Sergentomyia minuta*	**unreliable identification**	0.78	**unreliable identification**	0.67	No	*Ph. tobbi*	1.03	*Ph. perfiliewi*	1.02	Yes

Supplementary Table S2. Characteristic of specimen included in this study.

Species number	Species	Country	Collection sites	Collection date	Sex	Note	Genebank accession number
MOF8	*Ph. perniciosus*	Morocco	Chefchaouene (Loubar)	July 23rd, 1995	male	atypical	OP617463
MOF10					male	atypical	OP617464
MOF24					female		OP617465
MARSI19			Ghamate (Sidi Bouyahya)	July 14th, 2005	male	atypical	OP617466
MARSI21					male	atypical	OP617467
MARSI34					male	atypical	OP617468
MARSI35					male	atypical	OP617469
MARSI41					male	atypical	OP617470
MARSI46					male	atypical	OP617471
IT07		Italy	Gargano (Monte San Angelo)	September 3rd, 1991	male		OP617472
IT11					male		OP617473
IT14					male		OP617474
IT22					male		OP617475
IT24					male		OP617476
IT08					female		OP617477
IT12					female		OP617478
IT13					female		OP617479
FR35		France	Touraine (Beauvallon)	July 28th, 2002	male		OP617480
FR47				July 25th, 2007	male		OP617481
FR48					male		OP617482
FR50					male		OP617483
ES07		Spain	Catalonia (Begues)	August 19th, 1999	female		OP617484
ES12			Saragossa (El Bourgo de_Ebro)	August 13th, 1997	male		OP617485
ES13					male		OP617486
ES15					male		OP617487
ES16					male		OP617488
ES08					female		OP617489
ES09					female		OP617490

ES18				female	OP617491
ES19				female	OP617492
ES25		Murcia (Verdolay)	August 8th, 1997	male	OP617493
ES29				male	OP617494
ES31				male	OP617495
ES30				female	OP617496
ES37		Andalusia (Turre)	July 29th, 1997	male	OP617497
ES39				male	OP617498
ES43				male	OP617499
ES45				male	OP617500
PT08	Portugal	Algarve (Bordeira)	July 9th, 1996	male	OP617501
PT09				male	OP617502
PT13				male	OP617503
PT01				female	OP617504
PT03				female	OP617505
PT04				female	OP617506
PT05				female	OP617507
PT07				female	OP617508
PT19				female	OP617509
PT20				female	OP617510
PT31		Alto Douro (Cheires)	July 25th, 1996	male	OP617511
PT33				male	OP617512
PT29				female	OP617513
MT01	Malta	Gozo (Zebbug)	July 20th, 1989	male	OP617514
MT02				male	OP617515
MT10				male	OP617516
MT11				male	OP617517
MT12				male	OP617518
MT13				male	OP617519
MT24				male	OP617520
MT04				female	OP617521
MT05				female	OP617522
MT06				female	OP617523
MT07				female	OP617524

MT23					female		OP617538
IT09	*Ph. neglectus*	Italy	Gargano (Monte San Angelo)	September 3rd, 1991	male		OP617525
IT15					male		OP617526
IT18					male		OP617527
IT21					male		OP617528
ES06	*Ph. ariasi*	Spain	Catalonia (Begues)	August 19, 1999	female		0P617529
ES26			Murcia (Verdolay)	August 8th, 1997	female		OP617530
PT02		Portugal	Algarve (Bordeira)	July 9th, 1996	female		OP617531
PT24			Alto Douro (Cheires)	July 25th, 1996	female		OP617532
PT25					female		OP617533
PT37					female		OP617534
FR43		France	Dordogne (Vaunac)	July 25th, 2007	male		OP617535
FR44					male		OP617536
MT09	*Ph. perfiliewi*	Malta	Gozo (Zebbug)	July 20th, 1989	male		OP617537
FR49	*Ph. mascittii*	France	Dordogne (Vaunac)	25-Jul-2007	female		OP617539
FR51			Dordogne (Vaunac)		female		OP617540
FR59			Franche-Comté (Seillières)	July 22nd, 2003	female		OP617541
ES40	*Ph. papatasi*	Spain	Andalusia (Turre)	July 29th, 1997	male		OP617542
ES41					male		OP617543
MT08	*Ph. similis*	Malta	Gozo (Zebbug)	July 20th, 1988	female		OP617544
MARTAO02	*Ph. longicuspis*	Morocco	Taounate	July 19th, 1995	male	lcus_haplotype	OP617545
MARTAO04					male	lcus_haplotype	OP617546
MARTAO06					male	lcus_haplotype	OP617547
MOZ13			Ouezzana (Kchila)	July 23rd, 1995	female	lcus_haplotype	OP617548
MOZ14					female	lcus_haplotype	OP617549
MARTAO10			Taounate (Aïcha)	July 19th, 1995	male	lcx_haplotype	OP617550
MOZ02			Ouezzana (Kchila)	July 23rd, 1995	female	lcx_haplotype	OP617551
MOZ03			Ouezzana (Kchila)	July 23rd, 1995	female	lcx_haplotype	OP617552
MOF22			Chefchaouene (Loubar)	July 23rd, 1995	female	lcx_haplotype	OP617553
